# The London University Course

**Published:** 1909-09-04

**Authors:** 


### THE LONDON UNIVERSITY COURSE.

The University confers the degrees of Bachelor of Medicine and Bachelor of Surgery (M.B., B.S.), Doctor of Medicine (M.D.) and Master of Surgery (M.S.).

The student who desires to take the degree of M.B. of the London University has to pass the University Matriculation Examination, held thrice yearly in January, September, and June. The matriculation is accepted as a preliminary examination for graduation or qualification in medicine, provided the candidate has taken Latin as one of the optional subjects, and formerly no other examination exempted from the matriculation so far as degrees of the University were concerned. Recently, however, the Senate has seen fit to rule that several other examinations may be accepted instead of the usual matriculation.

After matriculating the student must attach himself to one or other of the various medical schools, and devote himself during his first year to mastering the subjects—chemistry, biology, and physics—required for his preliminary scientific or first professional examination. It will take him a full year to work up these subjects. Many candidates instead of doing the preliminary scientific, work somewhat more widely, and attempt to take the Intermediate scientific, which, provided the candidate has passed in the requisite subjects, exempts from the Preliminary scientific, and at the same time enables the student to proceed to his B.Sc. degree if he chooses.

The student is at present admitted to the Intermediate examination, the subjects of which consist of anatomy, physiology, and pharmacology, two years after having passed Part I. of the Preliminary. Time spent in working at the subjects of anatomy and physiology is not recognised by the University until the student has passed the Preliminary. His second and third years are thus spent in work in the dissecting room and physiological laboratory. Here he will be able to work more broadly and more minutely at the subjects than the Conjoint student is able to do. The examination itself is of average difficulty. The candidate is required to dissect a part, and is examined *viva voce* and by written papers in each subject.

Having passed his "Inter.," the student begins clinical work. Two years of clinical study were formerly required, but now three years will be necessary, while two and a half years will be the minimum period required for the preliminary subjects. Thus the full course will in future occupy at least five and a half years from the time of matriculation.

The final is by no means a stiff examination; compared with the Preliminary examination it is even an easy one. Special attention must be paid to a few subjects. Thus the student will do well to keep himself acquainted with pathology and morbid anatomy; midwifery and forensic medicine, also, are subjects in which many candidates fail. The last examination is written, oral, and practical. The candidate is required to examine and report on selected cases, to do practical bacteriological work, and to be acquainted with the essentials of operative surgery, though actual operations on the dead subject are not demanded.

